# LISTEN UP (Locally Integrated Screening and Testing Ear aNd aUral Programme): a feasibility study protocol for a community pharmacy-based ear health intervention

**DOI:** 10.1186/s40814-021-00856-6

**Published:** 2021-06-14

**Authors:** Selina Maree Taylor, Alice Cairns, Efi Mantzourani, Beverley D. Glass

**Affiliations:** 1 Centre for Rural and Remote Health - Mount Isa, 100 Joan Street, Mount Isa, QLD Australia; 2 Centre for Rural and Remote Health - Weipa, 407 John Evans Drive, Trunding, QLD Australia; 3 Cardiff School of Pharmacy and Pharmaceutical Sciences, Cardiff University, Wales, UK; 4 Primary Care, NHS Wales Informatics Service, Cardiff, Wales, UK; 5 College of Medicine and Dentistry, James Cook University, Townsville, QLD Australia

**Keywords:** Community pharmacy, Rural and remote, Pharmacy practice, Scope of practice, Ear

## Abstract

**Background:**

Ear disease is a major cause of preventable hearing loss and is very common in rural communities, estimated to affect 1.3 million Australians. Rural community pharmacists are well placed to provide improved ear health care to people who are unable to easily access a general practitioner (GP). The purpose of this study is to apply an ear health intervention to the rural community-pharmacy setting in Queensland, Australia, to improve the management of ear disease. The aims are the following: (1) to evaluate the feasibility, potential effectiveness and acceptability of a community pharmacy-based intervention for ear health, (2) to evaluate the use of otoscopy and tympanometry by pharmacists in managing ear complaints in community pharmacy and (3) to evaluate the extended role of rural pharmacists in managing ear complaints, with the potential to expand nationally to improve minor ailment management in rural communities.

**Methods/design:**

This is a longitudinal pre- and post-test study of a community-pharmacy-based intervention with a single cohort of up to 200 patients from two rural community pharmacies. Usual care practices pertaining to the management of ear complaints will be recorded prior to the intervention for 8 weeks. The intervention will then be piloted for 6 weeks, followed by a 12 month impact study. Patients aged > 13 years presenting to the pharmacies with an ear complaint will be invited to participate. Trained pharmacists will conduct an examination including a brief history, hearing screening, otoscopy and tympanometry assessments. Patients will be referred to a general practitioner (GP) if required, according to the study protocol. Patients will complete a satisfaction survey and receive a follow-up phone call at 7 days to explore outcomes including prescribed medications and referrals. Pharmacists and GPs will complete pre- and post- intervention interviews. Patient, pharmacist and GP data will be analysed using descriptive statistics and thematic analysis for the qualitative data.

**Discussion:**

This study will demonstrate the implementation of a screening and referring ear health intervention in rural community pharmacy. Feasibility, potential effectiveness and acceptability of the intervention will be assessed.

**Trial registration:**

Australian and New Zealand Clinical Trial Registry Number: ACTRN12620001297910.

**Supplementary Information:**

The online version contains supplementary material available at 10.1186/s40814-021-00856-6.

## Background

Ear care is recognised as important for the health of the population [1]. Ear disease is increasing globally with the World Health Organisation (WHO) proposing that by 2050 we can expect 900 million people to have disabling hearing loss, twice that of 2019 [2]. In Australia, more than 1.3 million people are living with a hearing condition that could have been prevented [3]. In rural and remote communities, the prevalence rate of middle ear diseases is as high as 50% in children under 3 years of age, double the prevalence recognised by WHO as a ‘massive public health problem’ [1, 2]. As well as the health consequences, unmanaged ear disease correlates with poor educational, social and behavioural outcomes [1].

Access to trained health care providers and a lack of infrastructure and supplies have been recognised as major challenges to providing ear care internationally [1]. There is currently a shortage of health care workers in rural and remote communities able to provide ear health care, which is predicted to worsen in the future [4]. Despite these shortages, there have been a number of innovative models of care developed to utilise consistently accessible health care professionals such as pharmacists to improve ear care [5]. A scoping review of community pharmacist interventions in ear health identified eight studies, whereby pharmacists provided a targeted ear health service, including hearing screening (4 in Australia), an otoscopy pilot study (1 in England) and pharmacy-based ear clinics (1 in USA; 2 in England) [5].

Pharmacists are trusted and accessible health professionals, who are motivated to meet local community needs [6]. Internationally, rural pharmacists are providing innovative models of care and working at expanded scopes of practice to better meet health needs [7]. Pharmacists, consumers and health professionals living in rural and remote locations in Australia are supportive of pharmacists expanding their service delivery to improve patient outcomes [8–10]. Rural pharmacists in Australia work in a unique setting with complex patients and limited access to health services and the potential for them to improve ear health care is unknown. A new pilot programme was developed to explore the impact of a pharmacist ear care intervention on patient-related outcomes.

Pilot and feasibility studies are an important step in the development of successful interventions for health [11]. There is emerging acknowledgement of the value of pilot studies to better understand the conduct and applicability of an intervention to allow the results to be better applied to patient care [11].

This paper describes the research protocol of the pilot, LISTEN UP (Locally Integrated Screening and Testing Ear aNd aUral Programme), a rural community pharmacy-based intervention to improve the management of ear health in the community in Australia.

### Research aims

This study aims to: (1) explore the feasibility, potential effectiveness and acceptability of a community pharmacy-based intervention for ear health, (2) evaluate the use of otoscopy and tympanometry by pharmacists in managing ear complaints in community pharmacy and (3) evaluate the extended role of rural pharmacists in managing ear complaints, with potential to expand nationally to improve ear care minor ailment management in rural communities.

## Methods and design

### Study design and setting

This is a longitudinal pre- and post-design study of a community-pharmacy-based intervention piloted in two rural community pharmacies in Queensland, Australia. Co-design has been applied to this study with stakeholder, health professional, pharmacist and consumer perspectives from previous research utilised in conjunction with community consultation to inform the design of this study [8–10]. Prior to the intervention, participating pharmacies will collect usual care data for 8 weeks beginning November 2020. The intervention will then be piloted for 6 weeks at each pharmacy and then refinement and improvements will be made before the longitudinal impact study is conducted for 12 months.

### Ethics approval

This project has been approved by the Human Research Ethics Committee, James Cook University (Reference number: H8187).

### Pharmacies

#### Pharmacy eligibility criteria

Community pharmacies that meet the following criteria are eligible to participate as a study site:

- Participating pharmacists must hold unconditional registration with the Australian Health Practitioner Regulation Agency (AHPRA) [12].
- Maintain accreditation standards for quality assurance under the Quality Care Pharmacy Programme (QCPP) [13].
- Have a private counselling area within the pharmacy that is separated from the common pharmacy counter, where one-to-one consultations can be conducted.
- Have a high daily ‘walk-in customer’ number of more than 100 customers per day.
- Have suitable information technology including a computer with internet access, printer and scanner.
- Are classified as rural or remote by the Modified Monash Model classification system categories 4-7 [14].
- Are located in Queensland, Australia, due to COVID-19 interstate restrictions around travel for training.

#### Recruitment of pharmacies

Pharmacies who have participated in earlier research on rural expanded pharmacy practice will be invited to express an interest to participate in the LISTEN UP. Those pharmacies who are interested will be phoned by the principal investigator to provide further explanation of the study and obtain consent. Two pharmacies will be enrolled in the study. Each pharmacy will be linked with at least one participating general practitioner. An invitation to participate with an information sheet and informed consent form will be provided to each pharmacist at the participating pharmacies and each GP at the participating general practices.

#### Pharmacist training

Each participating pharmacist will undertake nationally credentialed training in ear health including otoscopy and tympanometry. This training will be mixed mode with online and face-to-face components. The training includes 55 h of online training and two full days of workshops and is provided by the Benchmarque Group [15]. The training will include the following units of competencies: EHHPEH002—promote, educate and manage ear health; EHHAEH001—assess ear health; EHHPEA004—paediatric and TYMPTY001—perform tympanometry.

Only pharmacists who have successfully completed the required training will be eligible to participate in the study. Completed certificates of training will be provided to the principal investigator.

All training, including training materials will be consistent with national standards and will be tailored to suit the needs of community pharmacists. In addition, pharmacists will be provided with a list of recommended supplemental readings and resources. A member of the research team who is a pharmacy academic will also provide face-to-face and virtual training to the pharmacists on documentation processes for the project.

### General practitioners (GPs)

#### General practitioner eligibility criteria

GPs that meet the following criteria are eligible to participate in the study:

- Hold unconditional registration with the Australian Health Practitioner Regulation Agency (AHPRA).
- Have capacity to provide timely appointments (within 48 h) for participants referred to them for review.
- Have suitable information technology provisions including a computer with internet access, printer and scanner.
- Are classified as rural or remote by the Modified Monash Model classification system categories 4-7 [14].
- Are located in Queensland, Australia, due to COVID-19 interstate restrictions around travel for training.

#### Recruitment of GPs

At each pharmacy location, all GP practices within a 25-km radius will be invited to participate in the study.

### Participants

#### Sample size

The sample size was calculated using the formula *n* = Z^2^*P* (1-*P*)/*d*^2^, where n=sample size, Z is the critical value of the normal distribution at α/2 for a confidence level of 95% where α is 0.05 and the critical value is 1.96, *P* = expected prevalence or proportion = 0.14 (14%) and d = precision = 0.05 (5%) [16]. To our knowledge, there is no published community pharmacy-based ear health interventions of similar nature, therefore no standard reference could be applied to accurately determine prevalence required to calculate the sample size. However, we have calculated a sample size based on data from the Australian Government Department of Health, which estimates 14% of Australians suffer from hearing loss [3]. Therefore, *n* = 185 + 10% for missing data = 203 participants.

Given the calculated sample size, it is expected that each of the two participating pharmacies would recruit 100 patients into the study during the impact study. The duration of the project will be extended for up to 12 months to ensure adequate patient participant numbers to power the study.

#### Recruitment of participants

Potential participants will be recruited from walk-in customers who present at participating pharmacies seeking advice or products for an ear complaint. Pharmacists will invite these patients to participate in the study, provide an information sheet (with verbal explanation), ensure patient meets eligibility criteria and completes an informed consent form. Informed consent obtained from study participants is in written form.

### Participant eligibility criteria

To be eligible for participation in the study, patients must:

- Be aged 13 years or older (to be able to independently provide informed consent, those between 13-16 years can consent for self or parent/guardian may provide consent).
- Be able to understand the English language at a level appropriate to provide informed consent (pharmacists will use professional judgement to determine if participants are able to provide informed consent).
- Attend a participating pharmacy as a ‘walk-in’ customer seeking help for an ear complaint.

Patient will be excluded from the study if they:

- Are < 13 years old
- Have inadequate health literacy or English language skills to provide informed consent
- Have obvious major trauma to the ear
- Are a high COVID-19 risk patient (e.g. travelled in a COVID-19 hotspot within 14 days)
- Have not consented

### Intervention participants

Participants' temperature will be measured in the waiting area, if > 37.5 Celsius COVID-19 precautions will be implemented and additional personal protection equipment (PPE) applied, including face mask, gloves and face shield. Pharmacists will conduct the consultation with eligible consenting participants in a private consultation space. Pharmacists will then document a brief history of the ear complaint including symptoms, duration and treatments tried by the patient on a template service summary document (Appendix 1) provided to them. Pharmacists will then examine the ears using otoscopy and tympanometry. If the complaint is hearing related, pharmacists will perform a hearing screening test using the *Sound Scouts* application [17]. *Sound Scouts* is an application based hearing check that can be used in persons over the age of 4 years to detect conductive hearing loss, sensorineural hearing loss and difficulties listening in noise [17].

### Equipment

The otoscope used in this study is the MedRx video otoscope. The tympanometer is the Amplivox Otowave 102. Hearing screening will be conducted using the *Sound Scouts* application with Senheiser HD 300 headphones.

### Patient data collection

Patient data collected includes full name, postcode, age, gender, allergies, medicines, medical conditions, pregnancy/breastfeeding status, temperature, brief history of the ear complaint including symptoms, duration and treatments tried by the patient, otoscopy, tympanometry and hearing screening findings/results. This information will be documented on the service summary record. This record will contain all the information collected by the pharmacists from the patient consultation. It was developed in consultation with an advisory group (consisting of stakeholder representatives from various organisations in the health sector), is formatted in Microsoft Office and is stored on a password protected hard drive.

### Protocol

**Fig. 1:**
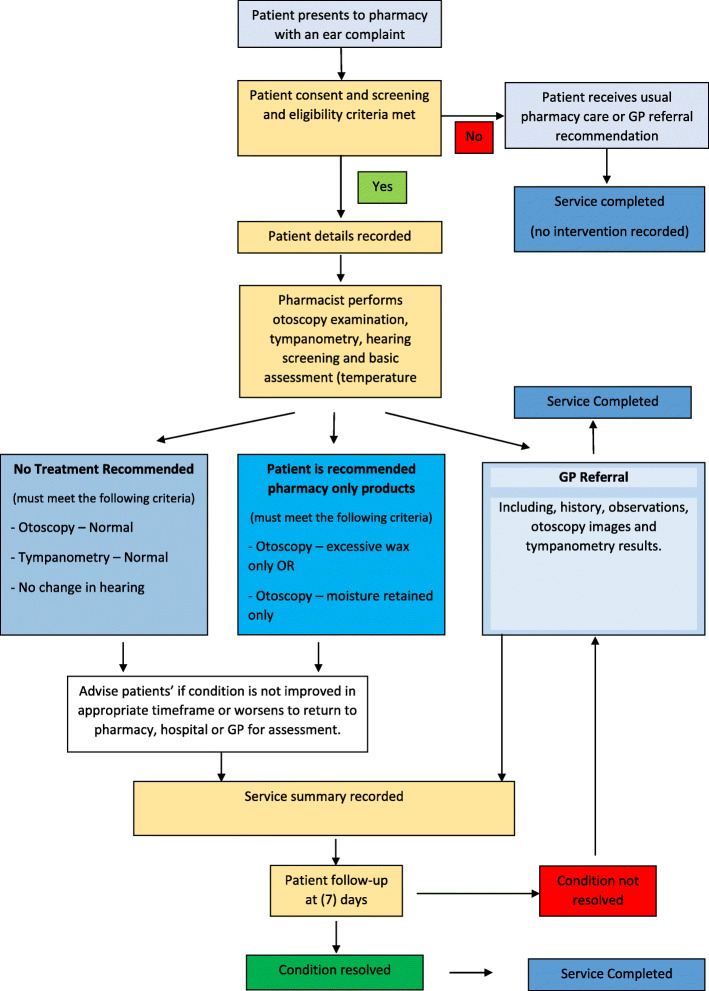
LISTEN UP study protocol pathway

Pharmacists will follow a protocol to determine the pathway (Fig. 1) for the patient. If otoscopy and tympanometry assessments are normal and hearing is not affected, the pharmacist may recommend no treatment and advise patient to monitor and seek medical advice if condition does not improve or worsens. If otoscopy indicates excessive wax only or moisture retention from water activity only and no other symptoms are present, the pharmacist may recommend pharmacy products including ear drops containing drying agents or wax dissolvents. All other patients will be referred to a GP with an appointment made by the pharmacist before they leave the pharmacy. Pharmacists will be able to book appointments with the GPs via a public online booking platform or via telephoning the GP practice. If the pharmacist is unable to make a timely appointment with a GP, the patient will be recommended to attend the local emergency department. Participants will be asked to complete a patient satisfaction survey and consent to a follow-up phone call in 7 days.

#### GP referral

The GP to which the patient has been referred will be emailed a password encrypted file with all of the patient data including temperature, brief history of the ear complaint including symptoms, duration and treatments tried by the patient, otoscopy, tympanometry and hearing screening findings/results.

#### Pharmacist recommendations

Pharmacists will be asked to record their actual recommendations and recommendations they would have made if they had an expanded scope including if they would have recommended a prescription medicine or referral to other service providers including audiometrists, speech pathologists, or ear, nose and throat specialists. This information will be collected for research purposes only as current practice does not allow Australian pharmacists to recommend prescription medicines or refer patients to specialty services.

### Follow-up

A member of the research team will phone all patient participants 7 days after their pharmacy consultation to explore the patient outcomes from the intervention. Patients will be asked about the condition of their ear complaint (improvement/deterioration), their satisfaction with the pharmacy intervention (Likert scale), if they were referred to a GP, if they attended the GP appointment and what advice, prescription or referral they had received from the GP. If the patient indicates further deterioration of the condition, a lack of improvement or a concern about the complaint, the researcher will offer to refer the patient to the GP and/or advise the patient to seek further medical advice.

### Data saturation

Total population sampling will be conducted in this study. We will attempt to interview all GPs and pharmacists by inviting them to participate in an interview three times. In addition, all participants will receive a follow-up phone call four times, including at least one out of normal business hours, in an attempt to ensure as many as possible participants receive the follow-up phone call.

### Study measurements and outcomes

Data pertaining to patient, pharmacist and GP experiences of the ear health intervention will be collected via semi-structured interviews pre- and post- intervention with pharmacists and GPs, service summary documentation, patient satisfaction surveys and 7-day follow-up interviews with patients. These data collection tools were developed in house to suit this innovative model. Pharmacist and GP interviews will include questions pertaining to perceptions of expanded pharmacy services, current local landscape of ear health (incidence, access to services) and expected/actual outcomes of the LISTEN UP project including pharmacist capacity, patient receptiveness and GP/pharmacist/patient interaction. Usual care data will be recorded for 8 weeks prior to the intervention. The usual care data will include a non-identifiable record of ear complaints presenting to the pharmacy, the description of the complaint and the pharmacists recommendations (Table 1).

**Table 1:** Data collection methods for pre-, during and post-intervention phases

	Patients	Pharmacists	General practitioners
Pre-intervention	Record of usual care in pharmacy for 8 weeks	Semi-structured interview	Semi-structured interview
During intervention	Patient satisfaction survey	Service summary document	
Post-intervention	Semi-structured interview (7-day follow-up)	Semi-structured interview	Semi-structured interview

Usual care data will record patient age groups, type of complaint (ear pain, ear wax, swimmers ear, ear itch, hearing loss or other), duration of the complaint, pharmacist recommendations (pharmacy products, verbal GP referral, verbal emergency department referral or other).

Initial study measurements are pharmacist and GP perspectives of ear health in the community, this described study protocol, expected outcomes, and anticipated enablers and barriers. This data will be collected prior to the study beginning via semi-structured interviews to explore the expected feasibility of the study. The interviews will be repeated post-study and the data collected from pre-intervention will be compared with data collected from these interviews to measure a change of opinion with pharmacists and GPs post-intervention.

Pharmacists will record the consultation data on a service summary document (Appendix 1). This document will also collect pharmacist recommendations for the patient, including they would have made if they had an expanded scope of practice such as prescription medicines and specialist referrals. This data will be compared with data provided by the patients at the 7-day follow-up phone call about the medicines they were prescribed and any referrals they may have received. In addition, qualitative data relating to the patient experience of the pharmacy service and patient perceived outcomes of the ear complaint will be collected during the patient interviews.

#### Study measurements

The study measurements collected in the intervention include pharmacist views, pharmacist recommendations, GP views and patient views. There measurements are aligned to the primary and secondary outcomes of the study (Table 2).

**Table 2:** Summary of study measurements aligned to study outcomes

Measurement	Instruments	Pre-study	During study	Post-study	Primary outcome	Secondary outcome
Pharmacist views	Semi-structured interview	X		X	1a	1, 2
Pharmacist recommendations	Service summary document		X			1, 2, 3
GP views	Semi-structured interview	X		X	1c	1, 2
Patient views	Satisfaction Survey			X	1b	1, 2
Patient views	Semi-structured interview			X	1b	1, 2, 3

#### Study outcomes

The outcomes of this study will be assessed against the objective of implementing a rural community pharmacy-based ‘model of care’ to improve the management of ear complaints in the community.

##### Primary

1. To evaluate the feasibility, acceptability and potential effectiveness of a community pharmacy-based intervention for ear health by exploring:
  - Pharmacist views of:
    i. Pharmacist capacity and competence to provide the intervention (motivation, confidence, competence, experience of training, capacity (workflow/workload))
    ii. Patient acceptance
    iii. Pathway to GP service (timeliness of appointment, GP staff attitudes)
  - Patient views of the service in terms of access, alternative health care options, satisfaction and willingness to pay (confidence/acceptance of pharmacist service, referral process, timeliness of pharmacists consult/GP consult).
  - GP views on appropriateness of pharmacist referrals, collaborative care with pharmacists (use of telehealth).
2. To evaluate the use of otoscopy and tympanometry by pharmacists to improve specificity of ear condition management in community pharmacy by comparing:
  - Usual care data with intervention data pertaining to pharmacist recommendations.
  - Pharmacist recommendations on the patient service summary record compared to GP prescriptions and referrals described by patients at the 7-day follow-up phone call.
  - Patient acceptance of pharmacists performing examinations with an otoscope and tympanometer.

##### Secondary

1. To evaluate the extended role of rural community pharmacists in managing ear complaints as a minor ailment in the community by evaluating, patient, GP and pharmacist perspectives of a community pharmacy-based ear health pre- and post-intervention.
2. To evaluate the potential for implementation of a national model of community pharmacy-based interventions to improve the management of minor ailments in rural communities.
3. To provide evidence to guide the scheduling of medicines to allow pharmacists to better manage minor ailments in community pharmacies.

### Data analysis

Data collected via semi-structured interviews will be transcribed verbatim and thematically analysed both inductively and deductively, using the NVivo 12 software programme [18, 19]. Data collected from the patient surveys and patient service summary record will be analysed using descriptive statistics and frequencies using IBM SPSS Statistics 25 for Windows.

## Discussion

The protocol and methods outlined will inform the development of an intervention framework for managing multiple minor ailments in the rural community pharmacy setting in Queensland, Australia. Positive outcomes from this study may demonstrate feasibility, potential effectiveness and acceptability of such an intervention. Internationally, expanded practice is becoming a common practice and is widely accepted in many countries; however, evidence to support expanded models of care in rural settings both internationally and in Australia are exceptionally limited and thus this protocol will add to the evidence base [7].

Preliminary discussions with professional pharmacy associations and professional indemnity insurers have been conducted and there is a high level of support for this programme.

### Limitations of the study protocol

This is a small pilot study of a complex intervention, with no control group. If the pilot testing indicates feasibility and effectiveness of this intervention, it will be important to validate the study with larger numbers in varied locations with a control group to comprehensively determine effectiveness and scalability. In addition, it was deemed out of scope for the small scale pilot protocol to include an economic evaluation of the study and thus a larger study would be required to examine economic sustainability.

## Conclusions

Ear disease is recognised as a major public health concern for rural and remote communities, especially due to accessibility of health professionals, requiring innovative strategies for effective management. Patients with ear complaints regularly present to community pharmacies seeking help due to difficulty in accessing GPs outside of metropolitan locations. Currently, pharmacists provide recommendations based on symptomatic descriptions of ear complaints provided by patients. Pharmacists are in an appropriately positioned location to provide improved ear care and are well placed to ensure patients are able to access timely health care. To our knowledge, this is the first community pharmacy-based study providing a specific ear health intervention in rural pharmacy practice to enable a pharmacist to improve the management of ear complaints.

## Supplementary Information

**Additional file 1.** Service summary document.

## Data Availability

The authors welcome any correspondence or requests for further details about this study protocol. The datasets used and/or analysed during the current study are available from the corresponding author on reasonable request.

## Abbreviations

GP: General practitioner
WHO: World Health Organisation
LISTEN UP: Locally Integrated Screening and Testing Ear aNd aUral Programme
AHPRA: Australian Health Practitioner Regulation Agency
QCCP: Quality Care Pharmacy Programme
PPE: Personal protective equipment

## References

[1] Macquarie University (2019). Hearing health in Aboriginal & Torres Strait Islander Peoples.

[2] World Health Organisation (2020). Deafness and hearing loss.

[3] Australian Government Department of Health (2020). Ear Health.

[4] Blazer DGDS, Liverman CT, Committee on Accessible and Affordable Hearing Health Care for Adults (2016). Hearing health care for adults: priorities for improving access and affordability.

[5] Taylor S, Cairns A, Glass B. Community pharmacist interventions in ear health: a scoping review. Primary Health Care Research & Development. 2021. (In Press). 10.1017/S1463423621000487 PMC8569911 34728002

[6] Pharmaceutical Society of Australia (2019). Pharmacist in 2023: for patients, for our profession, for Australia’s health System.

[7] Taylor S, Cairns A, Glass B (2019). Systematic review of expanded practice in rural community pharmacy. J Pharm Pract Res.

[8] Taylor S, Cairns A, Glass B (2021). Consumer perspectives of expanded practice in rural community pharmacy. Res Social Adm Pharm..

[9] Taylor S, Cairns A, Glass B (2020). Health professional perspectives of expanded practice in rural community pharmacy in Australia. Int J Pharm Pract.

[10] Taylor S, Cairns A, Glass B. Expanded practice in rural community pharmacy in Australia: pharmacists’ perspectives. J Pharm Pract Res. 2020; [Early View]. 10.1111/ijpp.12648 32602603

[11] Thabane L, Lancaster G. A guide to the reporting of protocols of pilot and feasibility trials. Pilot Feasibility Stud. 2019;5(37). 10.1186/s40814-019-0423-8. 10.1186/s40814-019-0423-8 PMC6393983 30858987

[12] Australian Health Practitioner Regulation Agency. 2021. https://www.ahpra.gov.au/. Accessed 30 Dec 2020.

[13] The Quality Care Pharmacy Program. 2021. https://www.qcpp.com/. Accessed 30 Dec 2020.

[14] Australian Government Department of Health (2019). Modified Monash Model Canberra: Australian Government Department of Health.

[15] The Benchmarque Group (2021). Ear and hearing health.

[16] Naing L, Winn T, Nordin R. Pratical issues in calculating the sample size for prevalence studies. Arch Orofac Sci. 2006;1:9–14.

[17] Sound Scouts HQ. Pty Ltd. Sound Scouts. 2020. https://www.soundscouts.com/au/about/evidence/. Accessed 30 Dec 2020.

[18] Fereday J, Muir-Cochrane E (2006). Demonstrating rigor using thematic analysis: a hybrid approach of inductive and deductive coding and theme development. Int J Qual Methods.

[19] QSR International (1999). NVivo Qualitative Data Analysis Software [Software].

