# Supplementary material for: LISTEN UP (Locally Integrated Screening and Testing Ear aNd aUral Programme): a feasibility study protocol for a community pharmacy-based ear health intervention
Source: Pilot Feasibility Stud. 2021 Jun 14;7:124. doi: 10.1186/s40814-021-00856-6 (PMC8200546; doi:10.1186/s40814-021-00856-6)
Supplement: Supplementary file 1 — Additional file 1. Service summary document. [file 40814_2021_856_MOESM1_ESM.docx]

# **SERVICE SUMMARY DOCUMENT**

Patient has received and reviewed information about the trial and research evaluation.

Patient has signed an informed consent form to participate in the trial and research evaluation.

Patient meets eligibility criteria to participate in the trial.

Date: __/___/____ Time: ___________

| Patient Contact Details | | | | | |
| --- | --- | --- | --- | --- | --- |
| First Name: |  | | Last Name: |  | |
| Address: |  | | | | |
| DOB: |  | | Gender: | Male/Female/Other | |
| Allergies: |  | | Medical Conditions: |  | |
| Pregnant? |  | | Breastfeeding |  | |
| Medications: |  | | | | |
| Episode of Care | | | | | |
| Presenting Complaint: |  | | | | |
| Duration of Complaint: |  | | Treatments tried: |  | |
| Pharmacist Examinations: | Otoscopy | Normal  Abnormal | | Tympanometry | Normal  Abnormal |
|  | Temperature: | |  |  | |
| Brief Notes: |  | | | | |

Attach images and results

| Pharmacists clinical impression: Eg. Otitis externa, wax impaction | |
| --- | --- |
|  | |
| Recommendations Made | |
| Pharmacist Recommendations | No treatment  Pharmacy-based treatment (please specify:________________________)  Referral with appointment made to GP  Other (please specify:________________________) |
| Expanded Practice Recommendations [RESEARCH PURPOSES ONLY] | |
| Prescription-only medicine (please specify exact drug/strength/dose: __________________________)  Immediate emergency department referral  Specialist ENT Referral  Speech Therapy Referral  Audiometry Hearing Test Referral  Other (please specify:________________________) | |

Time completed: _______________
